# Serum amyloid a protein as a potential biomarker in predicting acute onset and association with in-hospital death in acute aortic dissection

**DOI:** 10.1186/s12872-019-1267-0

**Published:** 2019-12-03

**Authors:** Yuchen He, Changcheng Ma, Jia Xing, Shiyue Wang, Chao Ji, Yanshuo Han, Jian Zhang

**Affiliations:** 1 Department of Vascular Surgery, the First Hospital of China Medical University, and Key Laboratory of pathogenesis, prevention and therapeutics of aortic aneurysm Liaoning Province, No. 155 Nanjing Bei Street, Shenyang, 110001 China; 2 Department of Clinical Laboratory, Shengjing Hospital of China Medical University, Shenyang, China; 3 Department of Histology and Embryology, China Medical University, Shenyang, China; 4 Department of Clinical Epidemiology, Shengjing Hospital of China Medical University, Shenyang, China; 5 Department of General Surgery, Shengjing Hospital of China Medical University, No. 36 Sanhao Street, Shenyang, 110004 China; 6 School of Life Science and Medicine, Dalian University of Technology, No. 2 Dagong Road, Liaodongwan New District, Liaoning, 124221 China

**Keywords:** Serum amyloid A, Acute aortic dissection, Biomarker, Inflammation

## Abstract

**Background:**

Acute aortic dissection (AAD) is a life-threatening disorder in vascular surgery with a high early mortality. Serum amyloid A (SAA) is a kind of acute-phase protein with a rapid diagnostic value in other diseases. However, the researches on the performance of SAA for the diagnosis of AAD is still lacking. This retrospective study aimed to evaluate the SAA levels and further explore its potential diagnostic role in AAD patients.

**Methods:**

SAA levels were measured by enzyme-linked immunosorbent assay (ELISA) in 63 controls and 87 AAD patients. Laboratory examinations were also performed. And relative clinical information was collected from participants included in this study.

**Results:**

SAA levels were significantly higher in AAD patients than those in healthy controls. SAA levels were independently associated with the risk of AAD. There was a positive significant correlation between SAA and C reactive protein (*R* = 0.442, and *P* = 0.001). Based on receiver-operating characteristic (ROC) analysis, the area under the curve (AUC) of SAA for the diagnosis of AAD were 0.942 with optimal cut-off points of 0.427 mg/L. For in-hospital mortality, the AUC of SAA were 0.732 with optimal cut-off points of 0.500 mg/L. According to logistic regression analysis, higher SAA levels represent a higher risk of in-hospital mortality (OR = 1.25; 95%CI: 1.07–1.47; *P* = 0.005).

**Conclusion:**

Our findings demonstrated that SAA levels were significantly enhanced in AAD. SAA was closely correlated with inflammatory parameters and coagulation-related parameters in AAD. Furthermore, SAA could be a potential bio-marker for identifying AAD in the early diagnosis. Finally, SAA > 5.0 mg/L are independently related to AAD in-hospital mortality.

## Background

Acute aortic dissection (AAD) is a life-threatening disorder in vascular surgery defined as the separation of aortic wall layer [1, 2]. In line with the Stanford classification, AAD is commonly divided into type A aortic dissection (TAAD) (involving ascending aorta) as well as type B aortic dissection (TBAD) (ascending aorta not affected) by considering both dissection extent and lesion site [3, 4].

AAD is characterized by acute onset, rapid progression as well as high morbidity and mortality in early stage. It has been previously reported that approximately 48.6% of untreated AAD patients die pre-hospitally [5]. Meanwhile, both TAAD and TBAD have high short-term in-hospital mortality. Medical imaging, including computed tomography (CT) and magnetic resonance imaging (MRI), is a reliable approach for AAD diagnosis [2, 6]. However, due to the mimic clinical manifestations between AAD and other common disorders and the uncertain aorta site of AAD lesion, it is difficult to diagnose on initial evaluation. Therefore, it is critically necessary to make early and confirmed diagnosis for patients with AAD to prevent the disease proceeding [7].

In addition, the clinical values of several serum biomarkers in the early diagnosis of AAD are increasingly recognized [8, 9]. For instance, C reactive protein (CRP) and D-dimer have been included in the ESC guideline for aortic diseases for the assessment of AAD patient conditions [10–12]. An ideal biomarker should be generally characterized by a rapid diagnostic value, a low cost, noninvasive and easy to perform with its potential sensitivity and specificity.

Serum amyloid A (SAA), is a specific apolipoprotein of high-density lipoprotein cholesterol (HDL-C), involved in the acute phase response. Diverse functions of SAA have been proved in cardiovascular diseases (CVD), including regulating matrix metalloproteinase-2 activity in aorta [13], modifying the vascular functionality of HDL-C [14], and regulation of systemic inflammatory reaction [15]. Recent research efforts have focused on the potential role of SAA as a biomarker in clinical disorders [16, 17]. However, there has been no study concerning the value of SAA in the early diagnosis and prognostic prediction in AAD.

In this study, the levels of SAA were assessed in AAD subjects as well as normal controls, followed by analysis of the potential correlation of SAA with AAD. We also investigated the diagnostic performance of SAA as a novel clinical biomarker in AAD prediction. Moreover, we also aimed to analyze the association between SAA and these classical biomarkers, including CRP as well as D-dimer level, followed by analysis on the possible combined diagnostic efficacy to detect AAD. Finally, the possible correlations between AAD prognosis and SAA level were identified.

## Methods

### Study population

**Fig. 1:**
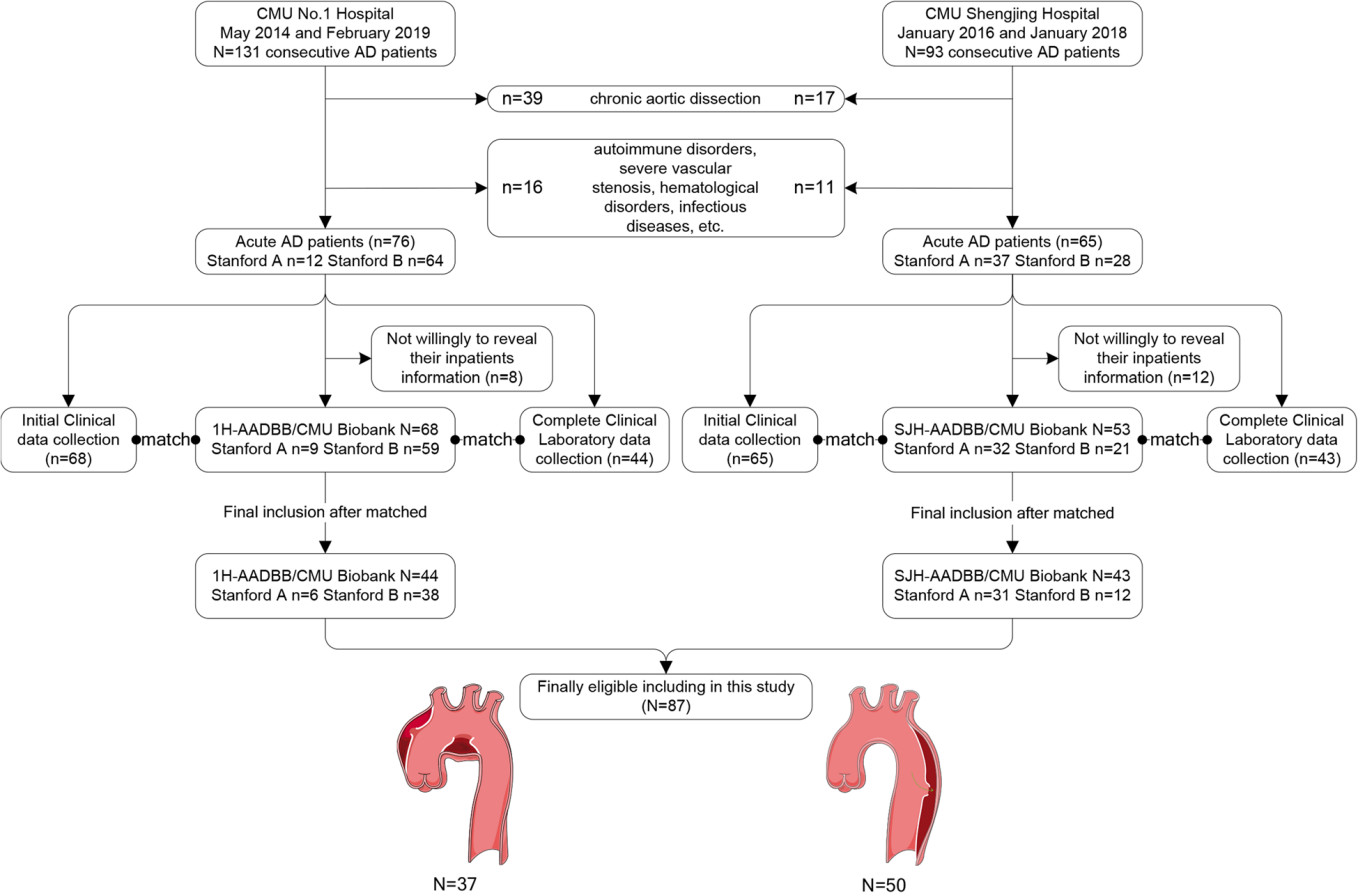
Flowchart of AAD patient selection and blood sample recovered from the First Hospital of CMU Aortic Dissection Blood sample Biobank (1H-ADBB/CMU) and Shengjing Hospital of CMU Aortic Dissection Blood sample Biobank (SJH-ADBB/CMU)

In this retrospective study, eligible aortic dissection patients were selected from two Affiliated Hospital of China Medical University (Shenyang, China). In brief, 131 consecutive AAD patients with corresponding peripheral blood samples and clinical data were collected from the Department of Vascular Surgery of the First Hospital of China Medical University (CMU) immediately after admission between May 2014 and February 2019. In addition, another 93 AAD patients were collected from the Department of Clinical Laboratory, Shengjing Hospital of CMU between January 2016 and January 2018. Among these 93 patients, 50 and 43 patients were diagnosed with TAAD and TBAD, respectively, during hospitalization (Fig. 1). The diagnosis of AD was based on imaging outcomes (CT, MRI and echocardiography). The AD was categorized in line with Stanford classification [18].

### Definitions

AD could be divided into acute as well as chronic phases. AAD is diagnosed if a patient is admitted to hospitalization within 14 days of symptom onset, otherwise, chronic AD is considered [5]. Moreover, acute AD patients admitted to the department of emergency of our two clinical centers were clearly diagnosed after careful assessment within 24 h following symptom onset. The diagnostic criteria of hypertension included a clinical record of systolic blood pressure (BP) ≥140 mmHg and/or diastolic BP ≥90 mmHg, and the administration of anti-hypertensive drugs. The definition of diabetes mellitus (DM) included fasting glucose level ≥ 7.0 mmol/L, glycosylated haemoglobin A1c ≥ 6.5% and the administration of oral hypoglycaemic drugs or insulin. In addition, the smoking status was judged in accordance with self-report current smokers.

The definitions of terms utilized in this research were listed in the following. The definition of a communicating false lumen in AAD is the opacification of at least partial false lumen with contrast media except ulcer-like projection [19]. On the contrary, a non-communicating false lumen in AAD is defined by the completely occlusive false lumen by a thrombus, as well as ulcer-like projection. Moreover, the definition of upper and lower strata SAA levels were set in line with cut-off value of receiver operating curve (ROC), which was 0.427 mg/L as cut-off value in the present study.

### Exclusion criteria

The AD patients with the following disorders were eliminated: chronic aortic dissection, malignancy, autoimmune disorders, severe aortic stenosis which was defined by an aortic valve area less than 1.0cm^2^ or less than 0.6cm^2^/m^2^ if indexed to body surface area [20], hematological disorders, infectious diseases, coronary artery disease which was defined as the presence of at least one≥50% stenosis in a coronary artery≥2.0 mm in diameter based on either coronary computed tomography angiography or invasive coronary angiography examination [21], severe organ failure, congenital heart disorders, previous aortic operation, Marfan syndrome, Ehlers-Danlos syndrome, other types of combined connective tissue or vascular disorders, and those receiving non-steroidal anti-inflammatory drugs or steroids. These disorders were ruled out using angiographic diagnosis, imaging examinations, laboratory tests, echocardiography, and other medical examinations based on the clinical presentations and medical history of patients. Eventually, there were 55 from First Hospital and 28 from Shengjing Hospital incongruent patients eliminated in this study (Fig. 1).

### Aortic dissection blood sample biobank

After excluding incongruent participates, on February 2019, eight and twelve patients were unwillingly to provide their inpatient record for publication, including their clinical data or blood samples, respectively (Fig. 1). Finally, aortic dissection blood samples from 68 patients were eligible for further analyses as Biobank, which registered in the First Hospital of CMU Aortic Dissection Blood sample Biobank (1H-ADBB/CMU) were selected to identity AAD. As a result, a total of 53 blood samples from AAD patients registered in Shengjing Hospital of CMU Aortic Dissection Blood sample Biobank (SJH-ADBB/CMU) were eligible and further enrolled in this study.

Venipuncture was conducted on eligible patients after their admissions, followed by sample collection of in EDTA plastic tubes (BD Vacutainer® lavender, 5.0 mL) and anticoagulant and silica/gel plastic tubes (SST BD Vacutainer® gold, 5.0 mL). Moreover, blood sample was centrifuged to collect plasma, which was reserved at − 80 °C for further test (up to 1 year). Peripheral blood mononuclear cells (PBMCs) of these AD patients were extracted by Ficoll-sodium diatrizoate density gradient centrifugation as described previously [22].

The present AD Biobank research was conducted in line with the Guidelines of the World Medical Association Declaration of Helsinki, and was approved by the Ethics Committee of Shengjing Hospital (Ethics Approval No. 2016PS085K) of China Medical University. All 121 subjects signed written informed consent.

### Serum measurements

ELISA kits (SAA: Wuhan Boster Biotechnology Company, China) were purchased to determine the SAA levels according to the manufacturer’s protocol.

### Laboratory examinations

#### Lipid panel

The plasma levels of low-density lipid cholesterol (LDL-C) and high-density lipid cholesterol (HDL-C) were directly detected using selective solubilization method (LDL-C test Kit or Determiner L HDL, Kyowa Medex, Tokyo). Additionally, levels of total cholesterol (TC) as well as TG were measured by enzymatic methods. Automatic biochemistry analyzer (ARCHIRECT ci16200, Abbott Laboratories, USA) was utilized to produce lipid profiles.

#### Additional biochemistry

Alanine aminotransferase (ALT) and glutamic oxalacetic transaminase (AST) were determined utilizing International Federation of Clinical Chemistry approach (Abbott Laboratories, USA). The plasma concentration of total protein (TP) was determined using biuret method (FUJIFILM Wako Pure Chemical industries Ltd., Japan). The fasting plasma glucose (FPG) levels were determined by urease GLDH and glucose oxidase methods (DiaSys Diagnostic Systems GmbH, Germany).

#### D-Dimer and CRP

Immunoturbidimetry was used to assess plasma D-dimer levels (Diagnostica Stago, France, normal limit ≤0.5 μg/mL). High-sensitivity assay with BN II nephelometer (Dade Behring, Germany) was used to detect CRP levels (normal limit ≤0.17 mg/L).

All blood analyses were carried out by Department of Clinical Laboratory at CMU Shengjing Hospital and Department of Clinical Laboratory of First Hospital of China Medical University for SJH-AADBB/CMU Biobank and 1H-AADBB/CMU Biobank, respectively. Follow by clinical data collection, blood Biobank establishment, and laboratory examinations, total of 87 eligible participates (TAAD = 37 and TBAD = 50) were finally identified to meet the inclusion criteria. A summary of the flow of participants’ selection and inclusion process is illustrated in Fig. 1.

### Control group (ctrl)

The blood sample from 87 patients with AAD were available from the venipuncture together with the blood samples from 63 matched controls. Shengjing Hospital of China Medical University Hospital medical examination database was used to identify healthy control by thoroughly searching all patients admitted to the emergency department diagnosed with trauma or motor vehicle accident during the 2014–2018 period (with imaging examinations, CT and/or MRI, at admission). All participants that were hemodynamicly stable and had non-typical symptoms, were considered as candidates of the control group. The control group was selected because these patients would not be expected to present any inherent bias favoring AAD. We also selected 20 matched patients diagnosed with stable angina as another control group. Stable angina was defined to chest discomfort that is classically retrosternal, triggered by exertion, and relieved by rest or nitrates within minutes [23]. Subjects diagnosed with vascular or connective tissue disorders using imaging examinations, CT and/or MRI, at admission were excluded from the control group. Other exclusion criteria included malignancy, infection, drug history, or any other immune-related disorders. Eventually, 63 participants in the control group and 20 patients in the angina group were included in this study. And all 83 subjects as control also signed written informed consent and conducted in line with the Guidelines of the World Medical Association Declaration of Helsinki.

### Statistical analysis

SPSS 22.0 (SPSS Inc., Chicago, IL, USA) was utilized for statistical analysis. Data were shown as medians with upper or lower quartiles for continuous variables due to the non-Gaussian data distribution. The difference between two groups was measured by non-parametric Mann-Whitney test, respectively. Comparisons among three groups were performed by one-way ANOVA, followed by Tukey’s post-hoc test, or nonparametric tests, followed by Kruskal-Wallis 1-way ANOVA test, according to the normality of the values. Categorical variables were shown as numbers with percentages, and the differences between two groups (for both biochemical and clinical parameters) was determined by Chi-square test. Correlations between continuous variables were analyzed through partial correlation analysis accounting for age, gender, and smoking. Furthermore, multiple logistic regression analysis was conducted to evaluate the correlation of serum SAA and D-Dimer or CRP with AAD risk following the adjustment of possible confounding factors. For instance, demographic characteristics and comorbidities were adjusted as confounding factors in all multivariable logistic regression models. Receiver operator characteristic (ROC) curves with area under the curve (AUC) along with logistic models were employed to determine the corresponding cut-off points and to assess the diagnostic performance of serum SAA and D-Dimer or CRP individually, and combined for AAD detection. *P* values < 0.05 were considered as statistical significance.

## Results

### Basic clinical characteristics of patients

**Fig. 2:**
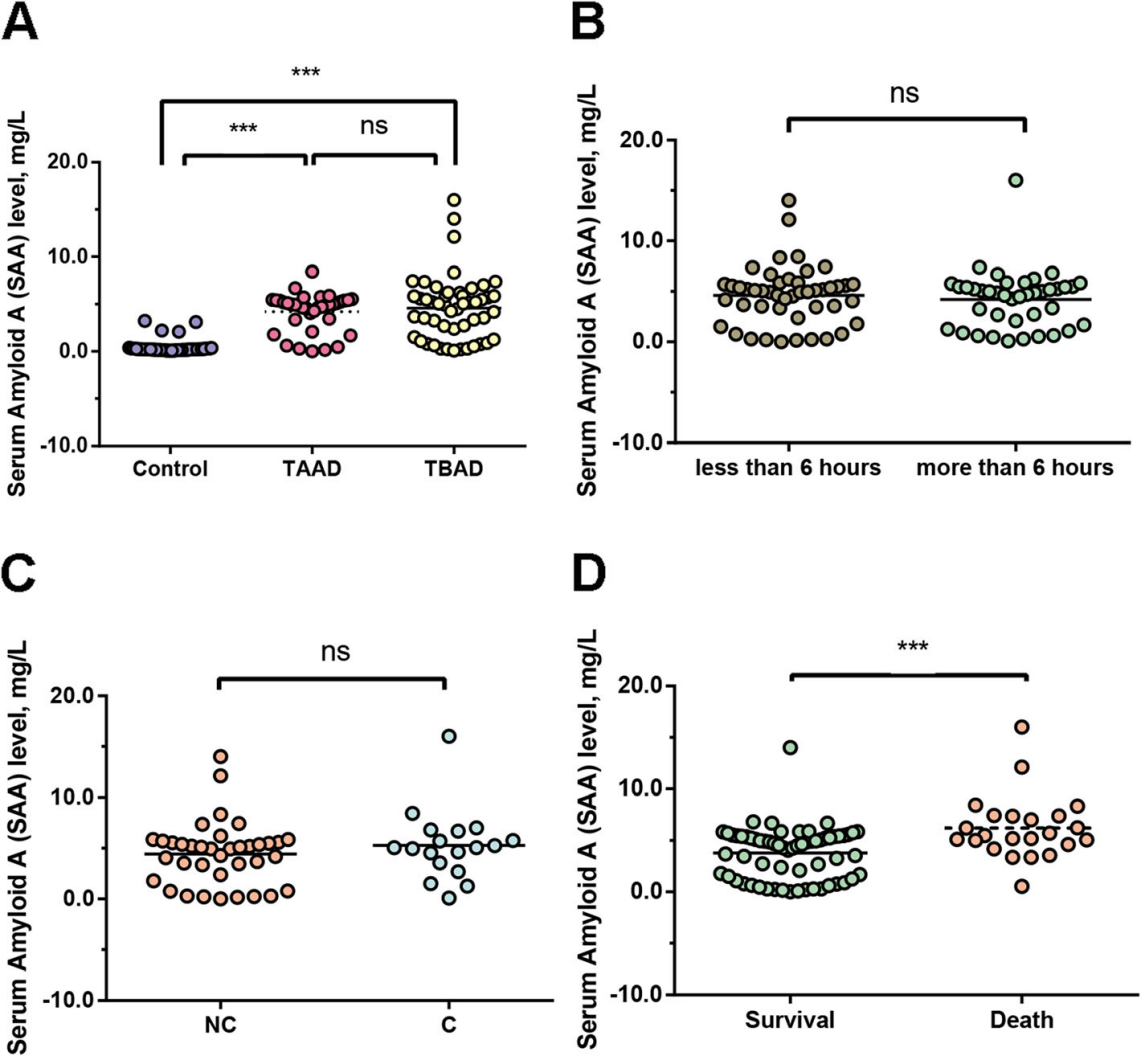
Analysis of SAA levels in different groups. **a** Comparison of SAA levels among controls, TAAD and TBAD. And sub-group analysis of SAA levels in AAD patients: **b** comparison of SAA levels between AAD with high-risk pain features that persisted less than six hours and more than six hours, and **c** comparison of SAA levels between AAD with communicating and non-communicating false lumen (C and NC), and **d** comparison of SAA levels between death cases and survival cases. *P < 0.05, **P < 0.01, ***P < 0.001, ns, no significance

The detailed clinical features of all subjects were shown in Table 1. Patients had significantly higher levels of heart rate, higher ratio of hypertension and smoker, higher white blood cell (WBC), platelet (PLT), and fast plasma glucose (FPG) (*P* < 0.001, *P* = 0.001, *P* = 0.048, *P* < 0.001, *P* = 0.033 and *P* < 0.001 respectively), but lower level of hemoglobin (Hb) in overall AAD, type A and type B groups in comparison to the control group (*P* < 0.001). Additionally, TAAD was not significantly different from TBAD in other comparisons. Nevertheless, these features were not statistically different between TAAD group and TBAD group. Moreover, in terms of other clinical features, including age, gender, BMI and rate of diabetes mellitus, healthy controls were not significantly different from AAD groups. Expectedly, the level of SAA protein was significantly enhanced in AAD group in comparison to the healthy control group (AAD vs Ctrl, *P* < 0.001; TAAD vs Ctrl, *P* < 0.001; TBAD vs Ctrl, *P* < 0.001; Table 1 and Fig. 2a). The SAA level was not significantly different between TAAD group and TBAD group (*P* = 0.595; Table 1 and Fig. 2a).

**Table 1:** Demographic and laboratory parameters of participants included in this study

	Control (*n* = 63)	Angina (*n* = 20)	AAD (*n* = 87)	*P*-value	TAAD (*n* = 37)	TBAD (*n* = 50)	*P*-value
				AAD vs Control			
				AAD vs Angina			
Male, n (%)	43 (68.25%)	15(75.00%)	65 (74.71%)	0.657	27 (72.97%)	38 (76.00%)	0.75
Hypertension, n (%)	21 (33.33%)	8(40.00%)	54 (62.69%)	0.002*	27 (72.97%)	27 (54.00%)	0.073
Smoking, n (%)	19 (30.16%)	7(35.00%)	31 (35.63)	0.774	12 (32.43%)	19 (38.00%)	0.594
DM, n (%)	10 (15.87%)	6(30.00%)	26 (29.89%)	0.123	11 (29.73%)	15 (30.00%)	0.978
Age, Yr	53.44 ± 11.52	57.80 ± 2.11	53.87 ± 11.42	0.957	53.32 ± 11.12	54.1 ± 11.73	0.848
				0.355			
BMI	21.8 ± 3.68	23.70 ± 0.73	22.87 ± 5.14	0.357	22.24 ± 3.92	24.02 ± 5.99	0.149
				0.702			
HR, bmp	78.06 ± 9.6	79.45 ± 1.68	89.46 ± 12.68	< 0.001***	90.49 ± 11.86	88.21 ± 13.28	0.273
				0.001***			
WBC, × 10^9^/L	5.83 ± 0.77	6.39 ± 0.40	10.74 ± 4.24	< 0.001***	11.54 ± 4.50	10.06 ± 3.92	0.198
				< 0.001***			
HGB, g/L	150.98 ± 17.68	135.50 ± 4.08	132.39 ± 20.97	< 0.001***	128.63 ± 17.86	135.61 ± 23.03	0.052
				0.297			
PLT, × 10^9^/L	173.3 ± 43.14	201.35 ± 13.27	190.59 ± 68.15	0.194	199.34 ± 67.05	183.12 ± 69.00	0.196
				0.744			
FPG, mmol/L	4.97 ± 4.87	5.54 ± 0.24	7.21 ± 2.42	0.001***	7.61 ± 2.84	6.92 ± 2.05	0.355
				0.155			
SAA, mg/L	0.36 ± 0.08	0.72 ± 0.40	4.43 ± 0.31	< 0.001***	4.22 ± 0.33	4.58 ± 0.48	0.595
				0.029*			

Note, *AAD* acute aortic dissection, *TAAD* type A aortic dissection, *TBAD* type B aortic dissection, *BMI* body mass index, *HR* heart rate, *DM* diabetes mellitus, *WBC* white blood cell, *HGB* hemoglobin, *PLT* platelet, *FPG* fast plasma glucose, *SAA* serum amyloid A. *P<0.05; **P<0.01; ***P<0.001

Additionally, we compared SAA levels between control subjects and AAD, angina patients and AAD patients stratified by cardiovascular risk factors (Table 2). SAA levels of AAD patients significantly elevated, compared with the control group and angina patients in each stratified analysis.

**Table 2:** Comparison of SAA levels between AAD and control group, AAD and angina group stratified by cardiovascular risk factors

Variables		Control	Angina	AAD	*P* Value	
		SAA (mg/L)	SAA (mg/L)	SAA (mg/L)	AAD vs Control	AAD vs angina
Age	<60Yr	0.418 ± 0.113	0.661 ± 0.333	4.436 ± 0.308	< 0.001	0.033
	≥60Yr	0.263 ± 0.084	0.632 ± 0.323	6.850 ± 2.535	< 0.001	0.023
Gender	male	0.375 ± 0.093	0.697 ± 0.295	5.542 ± 1.456	< 0.001	0.014
	female	0.332 ± 0.146	0.446 ± 0.244	5.227 ± 0.444	< 0.001	0.032
BMI	< 25	0.300 ± 0.067	0.652 ± 0.301	5.879 ± 1.496	< 0.001	0.017
	≥25	0.652 ± 0.316	0.630 ± 0.364	4.369 ± 0.494	< 0.001	0.016
Hypertension	Yes	0.445 ± 0.164	0.808 ± 0.204	4.892 ± 0.517	< 0.001	0.024
	No	0.319 ± 0.085	0.535 ± 0.342	6.397 ± 2.771	< 0.001	0.046
Smoking history	Yes	0.510 ± 0.215	0.728 ± 0.326	7.698 ± 2.990	< 0.001	0.006
	No	0.297 ± 0.063	0.516 ± 0.278	4.225 ± 0.344	< 0.001	0.030
DM	Yes	0.232 ± 0.093	0.566 ± 0.356	8.157 ± 3.569	< 0.001	0.007
	No	0.385 ± 0.093	0.677 ± 0.311	4.314 ± 0.317	< 0.001	0.047

Note, *AAD* acute aortic dissection, *BMI* body mass index, *DM* diabetes mellitus, *SAA* serum amyloid A

Sub-group analysis of AAD patient group was summarized in Table 3, without any significant differences between high-risk pain features that persisted less than 6 hours and more than 6 hours (*P* = 0.583; Fig. 2b). Additionally, there was no significant differences between and communicating and non-communicating false lumen (C and NC) for these clinical characteristics (*P* = 0.416; Fig. 2c). Thus, both survival and death cases were enrolled in the study subjects. As a result, WBC count (12.78 ± 4.05 vs 9.96 ± 4.08 × 10^9^/L, *P* = 0.004), percent of neutrophil (77.88 ± 10.9 vs 70.15 ± 14.72, *P* = 0.017), lactate dehydrogenase (826.64 ± 1148.14 vs 359 ± 255.33, *P* = 0.036), FPG (7.90 ± 2.25 vs 6.92 ± 2.46 mmol/L, *P* = 0.032), plasma D-dimer (6.20 ± 6.91 vs 1.83 ± 3.05 μg/mL, *P* = 0.006), CRP (138.21 ± 68.8 vs 48.78 ± 50.86 mg/L, *P* < 0.001) concentrations and SAA (6.214 ± 0.651 vs 3.783 ± 0.314 mg/L, *P* = 0.001) were significantly increased in death cases compared to those in survival cases (Fig. 2d).

**Table 3:** Comparisons of blood parameters between different sub-groups in AAD patient group

Parameters	TAAD	TBAD	*P* value	less than 6 h	more than 6 h	*P* value	communicating false lumen	non-communicating false lumen	*P* value	Death	Survival	*P* value
WBC, × 10^9^/L	11.54 ± 4.5	10.06 ± 3.92	0.198	10.73 ± 4.75	10.75 ± 3.78	0.917	12.1 ± 5.8	10.41 ± 3.74	0.522	12.78 ± 4.05	9.96 ± 4.08	0.004
BA/WBC, %	0.20 ± 0.23	0.28 ± 0.34	0.646	0.24 ± 0.3	0.25 ± 0.30	0.723	0.3 ± 0.28	0.23 ± 0.30	0.231	0.22 ± 0.21	0.26 ± 0.32	0.954
EO/WBC, %	1.37 ± 1.65	0.89 ± 1.51	0.139	0.95 ± 1.37	1.26 ± 1.76	0.409	1.04 ± 1.34	1.13 ± 1.65	0.783	0.66 ± 1.06	1.28 ± 1.72	0.039*
MO/WBC, %	13.29 ± 14.10	8.48 ± 9.03	0.002**	9.29 ± 7.37	11.96 ± 14.7	0.909	15.02 ± 19.28	9.63 ± 9.04	0.648	9.21 ± 9.16	11.26 ± 12.71	0.227
LY/WBC, %	14.87 ± 9.26	15.98 ± 9.1	0.462	14.76 ± 8.5	16.1 ± 9.72	0.596	14.24 ± 8.78	15.77 ± 9.26	0.514	11.62 ± 5.68	16.94 ± 9.79	0.043*
NE/WBC, %	69.92 ± 15.24	74.31 ± 12.97	0.198	74.49 ± 12.41	70.31 ± 15.41	0.275	69.4 ± 18.71	73 ± 12.86	0.638	77.88 ± 10.9	70.15 ± 14.72	0.017*
RBC, × 10^12^/L	4.26 ± 0.64	4.52 ± 0.76	0.014*	4.36 ± 0.65	4.43 ± 0.77	0.730	4.32 ± 0.80	4.42 ± 0.69	0.523	4.46 ± 0.82	4.38 ± 0.68	0.582
HGB, g/L	128.63 ± 17.86	135.61 ± 23.03	0.052	134.92 ± 18.44	130.13 ± 23	0.328	126.73 ± 27.9	133.79 ± 18.92	0.497	131.19 ± 25.29	132.85 ± 19.31	0.968
MCH, pg	30.45 ± 3.12	30.49 ± 1.94	0.373	31.06 ± 2.15	29.94 ± 2.75	0.015*	29.89 ± 4.59	30.61 ± 1.73	0.958	29.68 ± 3.41	30.77 ± 2.07	0.104
MCV, fL	91.66 ± 8.00	92.11 ± 5.52	0.696	93.2 ± 5.67	90.74 ± 7.43	0.181	90.10 ± 11.44	92.35 ± 5.01	0.681	89.83 ± 9.28	92.69 ± 5.36	0.168
MCHC, g/L	331.77 ± 11.11	331.12 ± 11.83	0.731	333.17 ± 9.78	329.85 ± 12.65	0.348	330.8 ± 17.09	331.57 ± 9.75	0.445	329.81 ± 11.29	332.04 ± 11.53	0.183
HCT, L/L	0.38 ± 0.06	0.41 ± 0.07	0.083	0.41 ± 0.06	0.39 ± 0.08	0.428	0.38 ± 0.09	0.40 ± 0.06	0.567	0.39 ± 0.08	0.40 ± 0.06	0.802
RDW, %	13.73 ± 2.43	13.25 ± 1.3	0.864	13.38 ± 1.47	13.51 ± 2.17	0.753	14.26 ± 2.64	13.25 ± 1.59	0.253	13.74 ± 1.78	13.33 ± 1.89	0.089
PLT, ×10^9^/L	199.34 ± 67.05	183.12 ± 69	0.196	177.75 ± 64.26	202.15 ± 70.25	0.218	166.07 ± 62.69	196.62 ± 68.56	0.168	182.33 ± 59.6	193.75 ± 71.4	0.597
MPV, fL	9.48 ± 1.13	9.93 ± 1.67	0.158	9.95 ± 1.43	9.52 ± 1.46	0.380	9.51 ± 1.77	9.77 ± 1.39	0.675	9.91 ± 1.06	9.65 ± 1.58	0.480
PCT, L/L	1.02 ± 2.26	0.36 ± 1.11	0.076	0.80 ± 1.99	0.56 ± 1.54	0.264	1.22 ± 2.57	0.55 ± 1.52	0.670	0.87 ± 2.10	0.59 ± 1.62	0.472
PDW, 10GSD	14.51 ± 2.72	14.4 ± 2.90	0.657	14.9 ± 3.00	14.06 ± 2.59	0.260	15.26 ± 2.12	14.27 ± 2.91	0.308	14.51 ± 2.53	14.42 ± 2.92	0.489
TP, g/L	63.93 ± 7.68	63.78 ± 10.34	0.540	64.76 ± 8.76	63.05 ± 9.56	0.410	61.72 ± 9.1	64.38 ± 9.19	0.277	62.27 ± 10.19	64.46 ± 8.77	0.591
PA, mg/dL	15.96 ± 6.67	17.71 ± 6.29	0.231	14.18 ± 6.55	18.88 ± 5.74	0.005**	16.94 ± 6.67	16.78 ± 6.52	0.868	14.75 ± 5.06	17.56 ± 6.83	0.177
ALB, g/L	35.75 ± 6.12	36.84 ± 6.93	0.341	37.03 ± 6.36	35.75 ± 6.74	0.470	34.45 ± 7.3	36.82 ± 6.34	0.185	36.63 ± 7.29	36.23 ± 6.32	0.813
ALP, U/L	106.79 ± 81.75	99.61 ± 43.41	0.583	104.84 ± 59.66	100.79 ± 66.18	0.793	102.42 ± 62.94	102.83 ± 63.19	0.925	103 ± 59.7	102.66 ± 64.29	0.773
ALT, U/L	140.94 ± 407.11	57.24 ± 61.02	0.151	91.74 ± 203.78	98.2 ± 333.43	0.210	119.07 ± 305.66	89.22 ± 274.01	0.408	147.86 ± 454.6	74.7 ± 170.15	0.224
AST, U/L	94 ± 364.89	60.56 ± 101.49	0.249	59.69 ± 101.85	91.36 ± 346.71	0.502	43.86 ± 45.18	83.97 ± 288.02	0.709	170.45 ± 488.11	41.54 ± 56.58	0.123
GGT, U/L	128.38 ± 212.94	94.02 ± 122.55	0.766	139.77 ± 181.81	83.2 ± 154.87	0.057	127.2 ± 192.56	105.2 ± 164.37	0.942	99.95 ± 158.57	113.35 ± 174.47	0.402
CHE, 1000 U/L	6.02 ± 1.65	5.96 ± 1.71	0.666	5.70 ± 1.54	6.22 ± 1.74	0.247	5.48 ± 1.68	6.13 ± 1.65	0.273	5.83 ± 1.54	6.05 ± 1.72	0.599
TBA, μmol/L	4.76 ± 4.87	4.16 ± 5.86	0.312	5.84 ± 7.11	3.36 ± 2.95	0.170	4.19 ± 3.29	4.54 ± 5.79	0.873	3.15 ± 2.67	4.95 ± 5.97	0.415
DBIL, μmol/L	8.93 ± 9.78	5.77 ± 2.84	0.493	9.7 ± 10.4	5.66 ± 3.36	0.122	8.72 ± 7.26	7.31 ± 8.06	0.463	6.58 ± 3.14	7.95 ± 8.77	0.815
TBIL, μmol/L	17.95 ± 14.17	16.75 ± 8.70	0.568	20.2 ± 14.67	14.81 ± 6.88	0.103	18.37 ± 10.52	17.01 ± 11.68	0.389	16.06 ± 8.68	17.74 ± 12.29	0.742
CK, U/L	186.1 ± 220.13	382 ± 1184.96	0.865	217.87 ± 314.36	360.08 ± 1186.69	0.940	287.88 ± 338.95	295.68 ± 974.92	0.122	562.31 ± 1711.62	203.15 ± 288.52	0.645
CKMB, U/L	17.95 ± 9.85	22.39 ± 22.55	0.343	18.16 ± 8.53	21.32 ± 21.17	0.930	18.23 ± 9.23	20.25 ± 17.83	0.914	26.84 ± 28.62	17.31 ± 8.18	0.607
Urea, mmol/L	7.56 ± 3.99	7.94 ± 8.41	0.127	7.84 ± 6.95	7.7 ± 6.56	0.640	8.29 ± 5.08	7.64 ± 7.08	0.296	7.87 ± 4.51	7.73 ± 7.41	0.646
Uric, mmol/L	273.5 ± 159.03	352.75 ± 214.01	0.280	332.93 ± 185.1	277.47 ± 184.43	0.213	314.57 ± 211.96	302.35 ± 179.47	0.623	376.29 ± 204.76	283.57 ± 175.97	0.177
Crea mmol/L	91.22 ± 43.83	111.17 ± 82.2	0.789	110.42 ± 66.54	88.13 ± 55.74	0.083	99.33 ± 70.69	98.91 ± 59.94	0.614	100.3 ± 22.1	98.58 ± 69.99	0.054
CYSC, mg/L	1.30 ± 0.53	1.42 ± 0.95	0.990	1.38 ± 0.79	1.34 ± 0.73	0.654	1.54 ± 0.84	1.32 ± 0.74	0.412	1.35 ± 0.63	1.36 ± 0.81	0.837
C1q, mmol/L	182.2 ± 35.56	200.05 ± 38.89	0.172	177.55 ± 36.91	198.33 ± 35.83	0.074	181.63 ± 33.57	191.32 ± 38.81	0.763	176.91 ± 31.95	191.81 ± 38.46	0.563
Na, mmol/L	138.48 ± 4.85	141.52 ± 6.29	0.037*	140.08 ± 7.12	140.15 ± 4.48	0.731	139.35 ± 8.79	140.3 ± 4.94	0.293	140.66 ± 6.7	139.91 ± 5.53	0.991
K, mmol/L	3.63 ± 0.87	4.27 ± 0.66	0.015*	3.92 ± 0.62	3.84 ± 1.02	0.724	4.18 ± 0.42	3.79 ± 0.91	0.095	3.7 ± 1.14	3.94 ± 0.74	0.274
CL, mmol/L	103.36 ± 5.92	111.18 ± 7.39	0.000***	104.75 ± 8.74	108 ± 5.91	0.052	105.88 ± 11.64	106.56 ± 6.14	0.875	108.57 ± 8.95	105.71 ± 7.01	0.704
HCO_3_, mmol/L	24.15 ± 2.41	23.98 ± 2.95	0.767	24.37 ± 2.47	23.76 ± 2.92	0.236	23.71 ± 2.39	24.14 ± 2.79	0.341	22.42 ± 2.71	24.69 ± 2.45	0.011*
PH value	6.93 ± 0.69	6.49 ± 1.08	0.106	7.06 ± 0.6	6.44 ± 1.02	0.021*	6.5 ± 1.04	6.81 ± 0.85	0.915	6.8 ± 0.81	6.73 ± 0.93	0.649
TC, mmol/L	4.19 ± 0.83	4.59 ± 1.17	0.255	4.62 ± 1.32	4.3 ± 0.84	0.589	4.92 ± 1.47	4.32 ± 0.93	0.361	4.51 ± 1.13	4.39 ± 1.03	0.333
TG, mmol/L	1.34 ± 0.56	1.34 ± 0.67	0.834	1.24 ± 0.47	1.41 ± 0.71	0.677	1.5 ± 0.57	1.31 ± 0.63	0.283	1.23 ± 0.44	1.39 ± 0.69	0.664
ApoA1, mmol/L	1.14 ± 0.18	1.21 ± 0.22	0.469	1.18 ± 0.17	1.18 ± 0.22	0.938	1.14 ± 0.31	1.19 ± 0.18	0.776	1.24 ± 0.16	1.14 ± 0.22	0.218
ApoB, mmol/L	0.85 ± 0.22	0.76 ± 0.13	0.674	0.75 ± 0.22	0.83 ± 0.13	0.277	0.85 ± 0.13	0.79 ± 0.18	0.506	0.76 ± 0.2	0.82 ± 0.16	0.425
LDL-C, mmol/L	2.58 ± 0.78	2.87 ± 1.06	0.246	2.87 ± 1.04	2.68 ± 0.92	0.620	3.23 ± 1.00	2.66 ± 0.94	0.133	2.65 ± 1.05	2.81 ± 0.93	0.899
HDL-C, mmol/L	1.08 ± 0.34	1.22 ± 0.31	0.139	1.18 ± 0.25	1.16 ± 0.37	0.649	1.09 ± 0.38	1.19 ± 0.31	0.570	1.25 ± 0.25	1.13 ± 0.35	0.150
LDH, U/L	451.88 ± 779.15	487.03 ± 489.75	0.138	505.85 ± 538.16	444.28 ± 694.87	0.171	410.67 ± 242.15	487.85 ± 692.2	0.645	826.64 ± 1148.14	359 ± 255.33	0.036*
INR	1.14 ± 0.22	1.08 ± 0.11	0.446	1.13 ± 0.21	1.09 ± 0.13	0.172	1.09 ± 0.12	1.11 ± 0.18	0.788	1.18 ± 0.26	1.08 ± 0.10	0.152
PT, s	13.11 ± 2.41	13.28 ± 1.33	0.149	13.51 ± 2.27	12.96 ± 1.51	0.119	12.9 ± 1.57	13.28 ± 1.96	0.634	13.91 ± 2.65	12.89 ± 1.36	0.131
PTA, %	85.87 ± 17.42	89.28 ± 12.04	0.615	85.62 ± 16.02	89.38 ± 13.59	0.198	88.54 ± 13.99	87.53 ± 15.01	0.974	83.4 ± 19.2	89.61 ± 12.04	0.279
APTT, s	32.28 ± 5.28	35.79 ± 5.86	0.008**	34.39 ± 6.11	34.04 ± 5.69	0.948	34.42 ± 6.43	34.14 ± 5.75	0.834	35.4 ± 6.4	33.67 ± 5.57	0.339
FPG, mmol/L	7.61 ± 2.84	6.92 ± 2.05	0.355	7.2 ± 1.92	7.22 ± 2.81	0.281	6.38 ± 1.63	7.45 ± 2.57	0.069	7.90 ± 2.25	6.92 ± 2.46	0.032*
CRP, mg/L	95.73 ± 60.92	70.53 ± 76.14	0.115	90.4 ± 83.01	71.92 ± 61.85	0.597	79.43 ± 84.49	79.81 ± 69.09	0.889	138.21 ± 68.8	48.78 ± 50.86	0.000***
D-Dimer, μg/mL	2.02 ± 3.57	4.10 ± 5.73	0.187	3.25 ± 4.94	3.09 ± 5.02	0.234	1.81 ± 1.69	3.49 ± 5.42	0.759	6.20 ± 6.91	1.83 ± 3.05	0.006**
SAA, mg/L	4.22 ± 0.33	4.58 ± 0.48	0.595	4.61 ± 0.42	4.21 ± 0.45	0.582	5.28 ± 0.81	4.44 ± 0.51	0.416	6.21 ± 0.65	3.78 ± 0.31	0.001***

Note, *BMI* Body Mass Index, *HR* heart rate, *WBC* white blood cells, *RBC* red blood cells, *HGB* hemoglobin, *PLT* Platelet, *BA* basophil, *EO* eosinophil, *LY* lymphocyte, *MO* monocyte, *NE* neutrophil, *MCH* mean corpuscular hemoglobin, *MCHC* mean corpuscular hemoglobin concentration, *MCV* mean corpuscular Volume, *MPV* mean platelet volume, *HCT* hematocrit, *PCT* platelet hematocrit, *PDW* platelet distribution width, *RDW* red blood cell volume distribution width, *APTT* activated partial thromboplastin time, *PT* prothrombin time, *PTA* prothrombin activity, *INR* international normalized ratio, *CK* creatine kinase, *CKMB* creatine kinase isoenzyme MB, *CRP* C-reactive protein, *ALB* albumin, *ALP* alkaline phosphatase, *ALT* alanine aminotransferase, *AST* aspartate aminotransferase, *DBIL* direct dilirubin, *TBIL* total bilirubin, *CHE* cholinesterase, *GGT* gamma glutamyl transpeptidase, *TBA* total bile acid, *TP* total protein, *PA* pre-albumin, *LDH* lactate dehydrogenase, *Na* serum sodium, *CL* serum chlorine, *Crea* Creatinine, *CYSC* Cystatin C, *K* serum kalium, *ApoA1* apolipoprotein A-1, *ApoB* apolipoprotein B, *FPG* fasting plasma glucose, *HDL-C* high-density lipoprotein-cholesterol, *TC* total cholesterol, *TG* triglycerides, *LDL-C* low-density lipoprotein-cholesterol

*P < 0.05; **P < 0.01; ***P < 0.001

### Association of SAA levels with clinical features

**Fig. 3:**
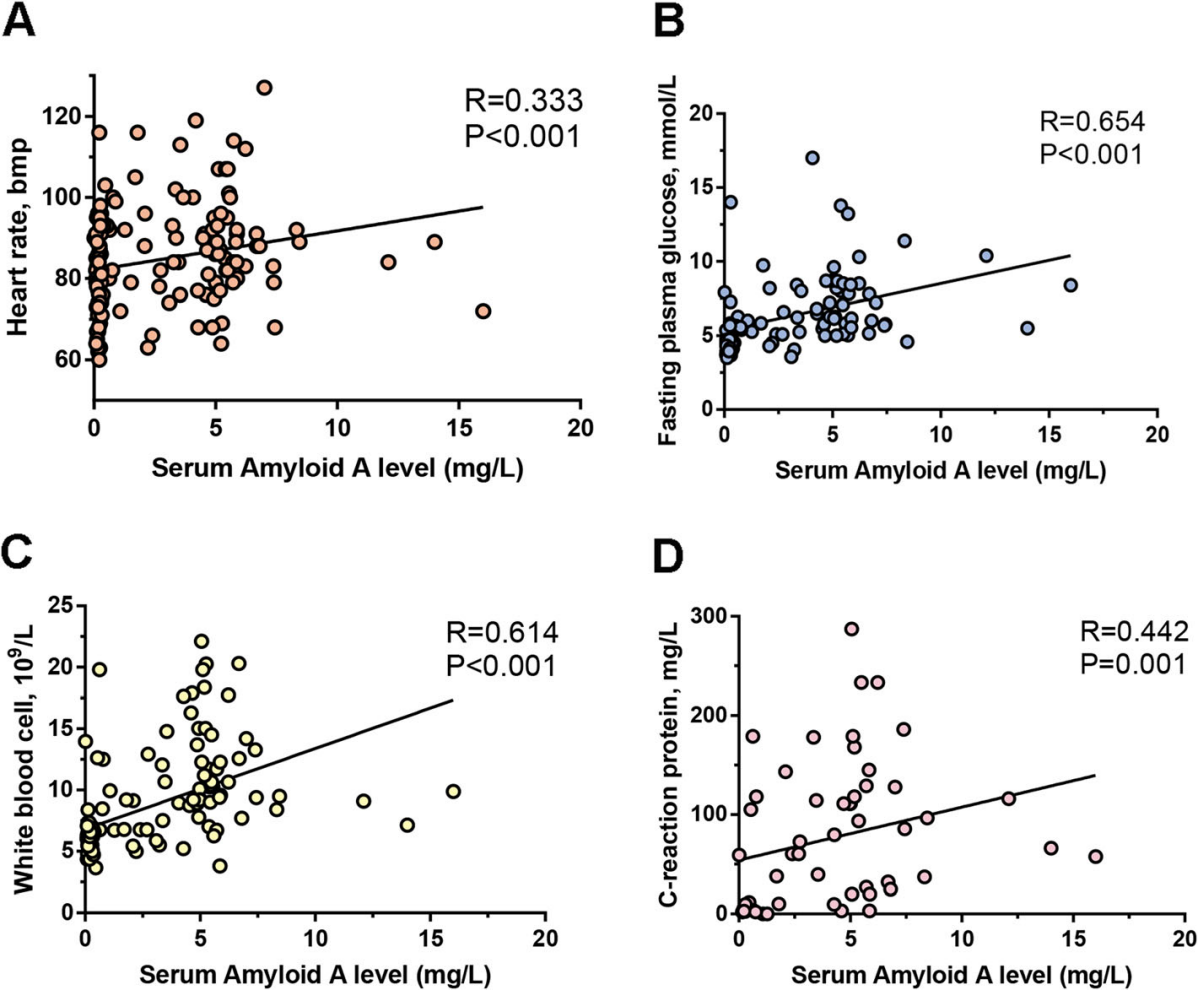
Correlations between SAA levels and clinical features in AAD patients. The horizontal axis represents SAA levels in mg/L and the vertical axis represents **a** heart rate, bmp, **b** Fast plasma glucose, mmol/L, **c** white blood cell, 10^9^/L, and **d** C-reactive protein, mg/L.

The correlation of SAA levels with clinical features was assessed in AAD subjects. As a result, SAA levels were positively correlated with heart rate (*R* = 0.333, and *P* = 0.001; Fig. 3a), FPG level (*R* = 0.654, and *P* < 0.001; Fig. 3b), WBC count (*R* = 0.257, and *P* = 0.042; Fig. 3c), lactate dehydrogenase (*R* = 0.357, *P* = 0.006), neutrophil count (*R* = 0.257, *P* = 0.025), Cystatin C level (CYSC, *R* = 0.248, *P* = 0.046) and plasma CRP concentration (*R* = 0.442, and *P* = 0.001; Fig. 3d). Correlations between SAA and other clinical characteristics were shown in Table 4.

**Table 4:** The correlations between SAA levels and clinical features in AAD patients

Characterization	Indices	R	*P* value
Baseline Data			
	Age, Yr	− 0.045	0.584
	BMI	0.126	0.123
	HR, bmp	0.333	< 0.001***
Blood routine			
	WBC, ×10^9^/L	0.614	< 0.001***
	RBC, ×10^12^/L	−0.111	0.354
	HGB, g/L	−0.395	< 0.001***
	PLT, ×10^9^/L	0.091	0.285
	BA, ×10^9^/L	−0.101	0.385
	EO, ×10^9^/L	−0.243	0.035*
	LY, ×10^9^/L	−0.168	0.147
	MO, ×10^9^/L	0.085	0.468
	NE, ×10^9^/L	0.257	0.025*
	MCH, pg	−0.065	0.574
	MCHC, g/L	−0.202	0.080
	MCV, fL	0.015	0.898
	MPV, fL	−0.098	0.405
	HCT, L/L	−0.198	0.148
	PCT, L/L	−0.011	0.930
	PDW, 10GSD	0.158	0.189
	RDW, %	0.171	0.166
Blood coagulation function			
	APTT, s	−0.241	0.051
	PT, s	0.03	0.811
	PTA, %	−0.258	0.037*
	INR	0.251	0.042*
	D-Dimer, ug/ml	0.206	0.097
Cardiovascular injury-related parameters			
	CK, U/L	−0.039	0.756
	CKMB, U/L	0.213	0.198
Inflammatory response			
	CRP, mg/L	0.442	0.001***
Liver function			
	ALB, g/L	−0.237	0.040*
	ALP, U/L	0.124	0.330
	ALT, U/L	0.121	0.300
	AST, U/L	0.163	0.166
	DBIL, umol/L	−0.098	0.553
	TBIL, umol/L	0.012	0.922
	CHE, 1000 U/L	−0.258	0.053
	GGT, U/L	0.028	0.811
	TBA, umol/L	0.138	0.310
	TP, g/L	−0.177	0.128
	PA, mg/dL	−0.402	0.002**
	LDH, U/L	0.357	0.006**
Renal function and serum electrolyte			
	Na, mmol/L	−0.141	0.224
	C1q, mmol/L	0.026	0.883
	HCO_3_, mmol/L	−0.282	0.020*
	CL, mmol/L	0.175	0.274
	Crea, mmol/L	−0.116	0.469
	CYSC, mg/L	0.248	0.046*
	PH value	0.113	0.523
	K, mmol/L	0.074	0.644
	Urea, mmol/L	−0.049	0.672
	Uric, mmol/L	0.182	0.336
Serum lipid profile			
	ApoA1, mmol/L	0.117	0.623
	ApoB, mmol/L	−0.117	0.623
	FBG, mmol/L	0.654	< 0.001***
	HDL-C, mmol/L	0.195	0.175
	TC, mmol/L	0.063	0.678
	TG, mmol/L	−0.223	0.120
	LDL-C, mmol/L	0.026	0.859

Note, *BMI* Body Mass Index, *HR* heart rate, *WBC* white blood cells, *RBC* red blood cells, *HGB* hemoglobin, *PLT* Platelet, *BA* basophil, *EO* eosinophil, *LY* lymphocyte, *MO* monocyte, *NE* neutrophil, *MCH* mean corpuscular hemoglobin, *MCHC* mean corpuscular hemoglobin concentration, *MCV* mean corpuscular Volume, *MPV* mean platelet volume, *HCT* hematocrit, *PCT* platelet hematocrit, *PDW* platelet distribution width, *RDW* red blood cell volume distribution width, *APTT* activated partial thromboplastin time, *PT* prothrombin time, *PTA* prothrombin activity, *INR* international normalized ratio, *CK* creatine kinase, *CKMB* creatine kinase isoenzyme MB, *CRP* C-reactive protein, *ALB* albumin, *ALP* alkaline phosphatase, *ALT* alanine aminotransferase, *AST* aspartate aminotransferase, *DBIL* direct dilirubin, *TBIL* total bilirubin, *CHE* cholinesterase, *GGT* gamma glutamyl transpeptidase, *TBA* total bile acid, *TP* total protein, *PA* pre-albumin, *LDH* lactate dehydrogenase, *Na* serum sodium, *CL* serum chlorine, *Crea* Creatinine, *CYSC* Cystatin C, *K* serum kalium, *ApoA1* apolipoprotein A-1, *ApoB* apolipoprotein B, *FPG* fasting plasma glucose, *HDL-C* high-density lipoprotein-cholesterol, *TC* total cholesterol, *TG* triglycerides, *LDL-C* low-density lipoprotein-cholesterol

*P < 0.05; **P < 0.01; ***P < 0.001

### Diagnostic performance of SAA, CRP and their combination for AAD

**Fig. 4:**
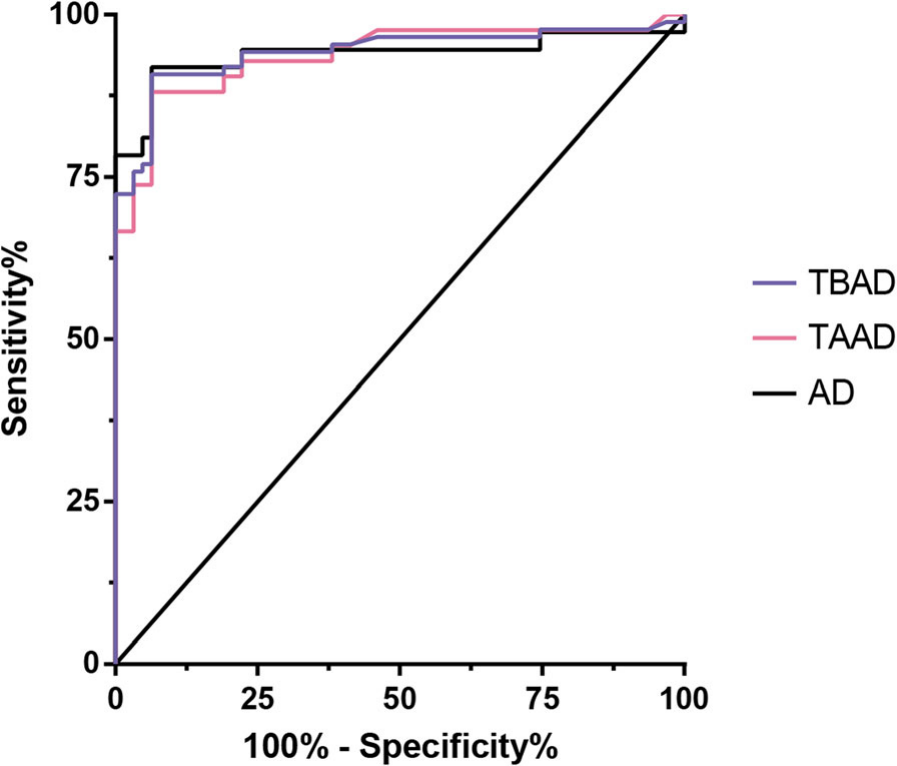
ROC analysis of SAA levels for the evaluation of TAAD, TBAD, and AAD. The vertical axis represents the sensitivity and the horizontal axis represents the 1-specificity

ROC analysis was conducted to determine the cut-off value of SAA level for the evaluation of TAAD, TBAD, and AAD. The AUCs for TAAD, TBAD, and AAD alone were 0.939, 0.937 and 0.942 with optimal cut-off points of 0.427 mg/L, 0.462 mg/L, and 0.427 mg/L, respectively, associated with sensitivity of 91.9, 88.1 and 90.8%, respectively, and specificity of 93.7, 93.7 and 93.7%, respectively (Table 5 and Fig. 4). Furthermore, the diagnostic performance of SAA, CRP and their combination to discriminate AAD according to ROC analysis, were also shown in Table 5. The AUC (0.977) of combined model (SAA + CRP) was significantly greater than that of SAA (AUC, 0.942), but not combined model (SAA + D-dimer, AUC = 0.900). Furthermore, SAA + CRP yielded sensitivity of 94.4% and specificity of 93.8%. ROC analysis was subsequently conducted to examine the cut-off values of SAA and other prognostic biomarkers for evaluation of in-hospital mortality (Table 5). The cut-off values were 4.998 mg/L for SAA, 82.55 mg/L for CRP, 1.945 μg/mL for D-dimer, and 72.976% for neutrophil to white blood cell ratio (NE%) with their higher sensitivity and specificity, respectively. The AUCs were 0.732 for SAA, 0.826 for CRP, 0.715 for D-dimer and 0.678 for NE%, respectively. In the case of SAA ≥4.998 mg/L, the sensitivity and specificity for in-hospital mortality prediction were 73.9 and 62.5% (95% CI 0.61 to 0.85; *P* = 0.001) for AAD patients. When CRP was ≥82.55 mg/L, the sensitivity and specificity were 0.789 and 0.771 (95% CI 0.709 to 0.942; *P*< 0.001). When D-dimer was ≥1.945 μg/mL, the sensitivity and specificity in predicting in-hospital death were 0.700 and 0.739 (95% CI 0.562 to 0.869; *P*= 0.006). When NE% was ≥72.976%, the sensitivity and specificity in predicting in-hospital death were 0.857 and 0.491 (95% CI 0.549 to 0.808; *P*= 0.017).

**Table 5:** Diagnostic value of SAA and its combinations with CRP and D-Dimer for AAD patients

	AUC	95%CI	P-value	cut-off	Sensitivity (%)	Specificity (%)
AAD	0.942	0.902–0.981	< 0.001	0.427	0.908	0.937
TAAD	0.939	0.873–1.004	< 0.001	0.427	0.919	0.937
TBAD	0.937	0.883–0.990	< 0.001	0.462	0.881	0.937
Combination						
SAA + CRP	0.977	0.955–0.999	< 0.001	–	0.944	0.938
SAA + D-Dimer	0.900	0.838–0.963	< 0.001	–	0.848	0.938
Death						
SAA (death v.s. survival)	0.732	0.612–0.853	0.001	4.998	0.739	0.625
CRP (death v.s. survival)	0.826	0.709–0.942	< 0.001	82.55	0.789	0.771
D-Dimer (death v.s. survival)	0.715	0.562–0.869	0.006	1.945	0.700	0.739
NE% (death v.s. survival)	0.678	0.549–0.808	0.017	72.976	0.857	0.491

Note, *AAD* acute aortic dissection, *TAAD* type A aortic dissection, *TBAD* type B aortic dissection, *SAA* serum amyloid A, *CRP* C reactive protein, *AUC* area under the curve, *NE%* neutrophil to white blood cell ratio

### The stratum of SAA according to gender and clinical biomarkers

The upper and lower strata of SAA levels were defined according to the cut-off values for the ROC, which in the present study were 0.427 mg/L for AAD patients. The prevalence of upper-stratum SAA levels was (78/87) 89.7% in AAD patients, 87.7% (57/65) in the male AAD group, and 95.5% (21/22) in the female AAD group. There were no significant gender differences of prevalence of upper stratum SAA levels (*P* = 0.438). Moreover, a multiple logistic regression analysis revealed that the plasma CRP, WBC, neutrophil, and HDL-C levels were positively associated with the upper stratum of SAA levels when compared with the lower stratum (OR = 1.05, 1.74, 1.97, 1.06, respectively, all *P*-values< 0.05). Furthermore, plasma pre-albumin and lymphocyte levels showed negative associations with the upper stratum of the SAA levels when compared with the lower stratum (Table 6).

**Table 6:** Associations of parameters with the upper stratum of the SAA levels when compared with the lower stratum

	SAA lower	SAA upper	*P* value
		OR (95%CI)	
Age, Yr	1 Reference	0.98 (0.92; 1.04)	0.534
Hypertension, n	1 Reference	3.12 (0.76; 12.84)	0.114
Smoker, n	1 Reference	1.64 (0.31; 8.54)	0.558
D-Dimer, μg/mL	1 Reference	1.72 (0.79; 3.75)	0.175
CRP, mg/L	1 Reference	1.05 (1.00; 1.09)	0.039
CAD, n	1 Reference	1.49 (0.17; 13.14)	0.719
Type01, n	1 Reference	1.11 (0.28; 4.50)	0.879
WBC, ×10^9^/L	1 Reference	1.74 (1.18; 2.56)	0.005
RBC, ×10^12^/L	1 Reference	0.55 (0.18; 1.66)	0.287
HGB, g/L	1 Reference	0.96 (0.92; 1.00)	0.071
PLT, ×10^9^/L	1 Reference	1.00 (0.99; 1.01)	0.974
FPG, mmol/L	1 Reference	1.17 (0.80; 1.70)	0.419
BA, ×10^9^/L	1 Reference	0.18 (0.03; 1.20)	0.076
EO, ×10^9^/L	1 Reference	0.79 (0.01; 114.81)	0.925
LY, ×10^9^/L	1 Reference	0.85 (0.77; 0.93)	0.000
MO, ×10^9^/L	1 Reference	1.02 (0.94; 1.10)	0.708
NE, ×10^9^/L	1 Reference	1.97 (1.24; 3.11)	0.004
CKMB, u/L	1 Reference	1.01 (0.89; 1.15)	0.836
MCH, pg	1 Reference	0.83 (0.56; 1.21)	0.332
MCHC, g/L	1 Reference	0.97 (0.91; 1.04)	0.432
MCV, fL	1 Reference	0.95 (0.84; 1.08)	0.459
MPV, fL	1 Reference	0.77 (0.47; 1.27)	0.306
PCT, L/L	1 Reference	0.51 (0.01; 3.93)	0.906
PDW, 10GSD	1 Reference	1.03 (0.80; 1.32)	0.829
RDW, %	1 Reference	2.43 (0.99; 5.97)	0.054
LDH, U/L	1 Reference	1.01 (1.00; 1.02)	0.063
ALB, g/L	1 Reference	0.94 (0.84; 1.05)	0.304
ALP, U/L	1 Reference	1.01 (0.99; 1.03)	0.383
ALT, U/L	1 Reference	1.01 (0.99; 1.02)	0.452
AST, U/L	1 Reference	1.00 (0.99; 1.02)	0.567
GGT, U/L	1 Reference	1.00 (1.00; 1.01)	0.529
DBIL, μmol/L	1 Reference	1.77 (0.71; 4.43)	0.223
TBIL, μmol/L	1 Reference	1.02 (0.94; 1.10)	0.617
PA, mg/dL	1 Reference	0.83 (0.71; 0.96)	0.010
TP, g/L	1 Reference	0.97 (0.89; 1.05)	0.396
TBA, μmol/L	1 Reference	0.97 (0.86; 1.10)	0.668
K, mmol/L	1 Reference	3.31 (0.91; 12.06)	0.070
CL, mmol/L	1 Reference	0.98 (0.81; 1.17)	0.801
Na, mmol/L	1 Reference	0.95 (0.86; 1.05)	0.313
HDL-C, mmol/L	1 Reference	1.06 (1.01; 1.10)	0.014
LDL-C, mmol/L	1 Reference	1.27 (0.52; 3.11)	0.605
TC, mmol/L	1 Reference	1.40 (0.56; 3.52)	0.471
TG, mmol/L	1 Reference	0.50 (0.15; 1.60)	0.240
Urea, mmol/L	1 Reference	1.17 (0.87; 1.55)	0.295
Uric, mmol/L	1 Reference	1.01 (0.98; 1.05)	0.389
Crea, mmol/L	1 Reference	1.09 (0.97; 1.23)	0.133
C1q, mmol/L	1 Reference	1.03 (0.98; 1.08)	0.264
HCO_3_, mmol/L	1 Reference	0.77 (0.57; 1.04)	0.086
PH value	1 Reference	0.08 (0; 921.56)	0.596
APTT, s	1 Reference	0.90 (0.80; 1.01)	0.079
PT, s	1 Reference	1.08 (0.71; 1.64)	0.724
PTA, s	1 Reference	0.96 (0.90; 1.02)	0.164

Note, *HR* heart rate, *WBC* white blood cells, *RBC* red blood cells, *HGB* hemoglobin, *PLT* Platelet, *BA* basophil, *EO* eosinophil, *LY* lymphocyte, *MO* monocyte, *NE* neutrophil, *MCH* mean corpuscular hemoglobin, *MCHC* mean corpuscular hemoglobin concentration, *MCV* mean corpuscular Volume, *MPV* mean platelet volume, *HCT* hematocrit, *PCT* platelet hematocrit, *PDW* platelet distribution width, *RDW* red blood cell volume distribution width, *APTT* activated partial thromboplastin time, *PT* prothrombin time, *PTA* prothrombin activity, *CKMB* creatine kinase isoenzyme MB, *CRP* C-reactive protein, *ALB* albumin, *ALP* alkaline phosphatase, *ALT* alanine aminotransferase, *AST* aspartate aminotransferase, *DBIL* direct dilirubin, *TBIL* total bilirubin, *CHE* cholinesterase, *GGT* gamma glutamyl transpeptidase, *TBA* total bile acid, *TP* total protein, PA pre-albumin, *LDH* lactate dehydrogenase, *Na* serum sodium, *CL* serum chlorine, *Crea* Creatinine, *CYSC* Cystatin C, *K* serum kalium, *ApoA1* apolipoprotein A-1, *ApoB* apolipoprotein B, *FPG* fasting plasma glucose, *HDL-C* high-density lipoprotein-cholesterol, *TC* total cholesterol, *TG* triglycerides, *LDL-C* low-density lipoprotein-cholesterol

### Univariate and multivariate logistic regression analyses for in-hospital mortality

The following ten variables were indicated to be related with hospital short-term mortality, WBC, eosinophils, lymphocyte, neutrophil, HCO_3_ level, lactate dehydrogenase, FPG, CRP, D-Dimer, and SAA concentration (*P* value was approximately 0.05 in univariate analysis) (Table 3). Therefore, the ten variables were incorporated into multivariate logistic regression analysis, revealing that SAA (OR = 1.25; 95%CI: 1.07–1.47; *P* = 0.005), CRP (OR = 1.03; 95%CI = 1.01–1.10; *P* < 0.001), WBC count (OR = 1.17; 95%CI: 1.03–1.33; *P* = 0.015) and eosinophils (OR = 1.06; 95%CI: 1.00–1.11; *P* = 0.041) were significantly related to hospital short-term mortality (Table 7) adjusted as confounding factors. Nevertheless, in-hospital death was not significantly associated with any of variables enrolled in this research.

**Table 7:** Univariate and multivariate logistic regression analyses for in-hospital mortality

	Model 1		Model 2	
	OR (95%CI)	*P*-value	OR (95%CI)	*P*-value
Type of AAD	0.47 (0.16; 1.32)	0.152	0.48 (0.17; 1.36)	0.167
HR, bmp	1.01 (0.97; 1.05)	0.584	1.01 (0.97; 1.05)	0.536
Hypertension, %	1.22 (0.41; 3.64)	0.724	1.13 (0.37; 3.40)	0.832
Smoker, %	0.63 (0.20; 1.96)	0.421	0.63 (0.20; 2.00)	0.432
D-Dimer, μg/mL	1.19 (0.98; 1.43)	0.074	1.20 (0.99; 1.46)	0.065
CRP, mg/L	1.02 (1.01; 1.03)	0.000	1.03 (1.01; 1.04)	0.000
WBC, ×10^9^/L	1.17 (1.03; 1.32)	0.013	1.17 (1.03; 1.33)	0.015
EO, ×10^9^/L	0.68 (0.40; 1.15)	0.148	0.68 (0.40; 1.16)	0.161
LY, ×10^9^/L	0.92 (0.85; 0.99)	0.030	0.92 (0.85; 0.99)	0.031
NE, ×10^9^/L	1.05 (1.00; 1.11)	0.040	1.06 (1.00; 1.11)	0.041
HCO_3_, mmol/L	0.69 (0.53; 0.89)	0.004	0.67 (0.51; 0.88)	0.004
LDH, U/L	1.00 (0.99; 1.00)	0.097	1.00 (0.99; 1.00)	0.151
FPG, mmol/L	1.17 (0.95; 1.44)	0.133	1.16 (0.95; 1.43)	0.149
SAA, mg/L	1.22 (1.05; 1.42)	0.011	1.25 (1.07; 1.47)	0.005

Note, *AAD* acute aortic dissection, *HR* heart rate, *CRP* C reactive protein, *WBC* white blood cell, *EO* eosinophil, *LY* lymphocyte, *NE* neutrophil, *LDH* lactate dehydrogenase, *FPG* fasting plasma glucose, *SAA* serum amyloid A, *CI* confidence interval, *Model 1* no adjustments, *Model 2* adjusted for age, gender, BMI

## Discussion

AAD is a severe aortic disorder associated with inflammation [8, 15, 24]. The inflammatory responses play a critical role in initiating further necrosis and apoptosis of smooth muscle cells as well as degeneration of elastic tissue, contributing to aorta rupture. SAA, an acute-phase protein, reflects the status of inflammation [25]. The early diagnostic value of SAA as a serum biomarker has been increasingly recognized in other diseases. To our knowledge, it was the first study to focus on the association between SAA and AAD, which demonstrated SAA as a potentially useful biomarker for AAD detection.

In this study, SAA levels were significantly higher in AAD patients than healthy controls. However, SAA levels were not significantly different between TAAD and TBAD, indicating that SAA was not associated with the specific subtype of AAD. Moreover, SAA was correlated with several laboratory examination outcomes, which were necessary to evaluate the status and to detect complications. According to the correlation analysis, SAA levels were significantly correlated with inflammation-related parameters, including WBC count, neutrophil count and CRP. CRP, an acute phase reactant, has been widely used as an independent predictor of poor prognosis in AAD patients, which is also related to the in-hospital mortality in AAD patients [12]. Additionally, SAA was also correlated with prothrombin activity and international normalized ratio in this study. AAD is widely known to be influenced by activated coagulation and fibrinolytic systems [26, 27]. Among these, D-dimer shows its important diagnostic and prognostic value in AAD patients [9]. However, SAA is not correlated with myocardial biomarkers in this study. The closely correlation between SAA and admission hematological parameters indicates the potential clinical significance of SAA in the clinical process of AAD.

Additionally, based on the elevated SAA levels in AAD patients, the diagnostic value of SAA alone and its combination with CRP as well as D-dimer was assessed for AAD detection. As a result, SAA was able to provide high sensitivity and specificity for TAAD diagnosis by referring to the ROC curves. However, our data revealed high specificity but relatively weak sensitivity of SAA when used alone for TBAD. Interestingly, compared with SAA alone, CRP-SAA combination led to improved diagnostic accuracy along with the increased sensitivity as well as higher AUC, while D-Dimer-SAA combination failed to show the significant elevation of AUC. Taken together, these findings indicated that SAA may be a potential non-invasive predictor of AAD, especially for AAD patients with normal CRP value.

Furthermore, SAA has been identified as an apolipoprotein of high-density lipoprotein cholesterol (HDL-C). Previous studies have indicated that HDL-C is negatively associated with cardiovascular risks, and low HDL-C levels may represent a high in-hospital mortality in AAD patients [28]. SAA-HDL complex in blood has been demonstrated to have a high affinity for macrophages [14] and to further eliminate them from the blood in patients with sarcoidosis [29]. Thus, increased SAA levels in AAD patients may be caused in response to the systemic inflammation, showing its potential value in predicting AAD characteristics. Additionally, on the one hand, the progressive degradation of extracellular matrix is also considered as a critical pathological feature in the pathogenesis and progression of AAD [30]. On the other hand, SAA has been reported to elicit several kinds of matrix metalloproteinases induction in vascular smooth muscle cells in vitro [15, 31, 32]. Thus, elevated SAA level in AAD is of great significance for the evaluation of AAD development. In this study, AAD patients with low SAA levels are more likely to control disease status than those with high SAA levels. Collectively, these outcomes suggest the potential prognostic value of SAA in the clinical procedure of AAD.

In this study, we observe that high SAA levels are associated with high in-hospital mortality. It has been previously reported that high SAA levels were associated with mortality in other diseases. For example, higher SAA concentration was associated with all-cause mortality in patients with end-stage renal disease [33]. Additionally, as an apolipoprotein of HDL, SAA can transforms HDL from a protective lipoprotein into a pro-atherosclerotic lipoprotein, which contributed to the substantially worsened cardiovascular outcome, which have been reported previously [34]. Our results suggest that SAA levels in AAA patients may be a critical marker of all-cause mortality in AAD. Additionally, it has been reported previously that CRP, D-Dimer [12] and NE% [35] are important risk factors for in-hospital mortality in AAD. In our study, we observed that CRP with AUC of 0.826, the highest sensitivity and specificity, is the best prognostic indicator of the mortality, and the AUC of SAA in predicting in-hospital mortality (AUC = 0.732) is better than that of D-Dimer (AUC = 0.715) and NE% (AUC = 0.678). Therefore, our result revealed the potential value of SAA as a prognostic biomarker of the in-hospital mortality in AAD.

However, there are some limitations in this study. To begin with, the population size was relatively small. Secondly, the specific study population was collected from only two clinical centers. Additionally, the detailed backgrounds of healthy controls were not clearly defined due to the incomplete recording of their previous medical history. Furthermore, a combination of tissue expression and mechanism study would definitely give rise to more detailed information concerning the role of SAA in AAD.

## Conclusions

Collectively, our findings demonstrated that SAA levels were significantly enhanced in both TAAD as well as TBAD patients. Then, we suggested the close correlations of SAA with inflammatory parameters and coagulation-related parameters in AAD patients at the level of serology. Furthermore, we provided the proof that SAA may be a useful biomarker for AAD identification, and SAA > 5.0 mg/L is considered as important risk factors, which are independently related to AAD in-hospital mortality. Nevertheless, prospective, large-scale clinical studies are warranted to further validate these outcomes, which would definitely contribute to the prognostic evaluation of AAD.

## Data Availability

The data used in this study are available from the corresponding author if needed.

## Abbreviations

1H-ADBB/CMU: The first hospital of CMU aortic dissection blood sample biobank.
AAD: Acute aortic dissection
ALT: Alanine aminotransferase
AST: Glutamic oxalacetic transaminase
AUC: The area under the curve
BP: Blood pressure
C and NC: Communicating and non-communicating false lumen
CMU: China Medical University
CRP: C-reactive protein
CT: Computed tomography
Ctrl: Control group
CVD: Cardiovascular diseases
CYSC: Cystatin C level
DM: Diabetes mellitus
FPG: Fasting plasma glucose
HDL-C: High-density lipoprotein cholesterol
LDL-C: Low-density lipid cholesterol
MRI: Magnetic resonance imaging
NE%: Neutrophil to white blood cell ratio
PBMCs: Peripheral blood mononuclear cells
ROC: Receiver-operating characteristic
SAA: Serum amyloid a
SJH-ADBB/CMU: Shengjing hospital of CMU aortic dissection blood sample biobank
TAAD: Type a aortic dissection
TBAD: Type B aortic dissection
TP: Total protein
WBC: White blood cell
